# A Cross-Sectional Population-Based Survey of Trachoma among Migrant School Aged Children in Shanghai, China

**DOI:** 10.1155/2016/8692685

**Published:** 2016-08-17

**Authors:** Wenwen Xue, Lina Lu, Jianfeng Zhu, Xiangui He, Jiangnan He, Rong Zhao, Haidong Zou

**Affiliations:** ^1^Shanghai Eye Disease Prevention & Treatment Centre, Shanghai 200040, China; ^2^Shanghai General Hospital, Shanghai Jiao Tong University School of Medicine, Shanghai 200080, China

## Abstract

We investigated the prevalence of clinical trachoma in 154,265 children aged 6 to 16 years in 206 Shanghai migrant schools. Clean water availability in school, each child's facial cleanliness, eyelids, corneas, and the presenting distance visual acuities were examined. Trachoma was clinically diagnosed in accordance with the World Health Organization simplified classification. Eyes diagnosed with trachoma were swabbed to test for ocular* Chlamydia trachomatis* infections (OCTI) with a rapid latex immunochromatographic test. Among 153,977 students, no blindness was found related to trachoma. Trachoma was diagnosed in 8029 children (5.2%). In 87 schools clinical trachoma prevalence was higher than 5%. OCTI was confirmed in 2073 of 6823 trachoma diagnosed children (30.4%). Clinical trachoma prevalence was higher among females than males (*p* < 0.001), but gender comparison showed no statistical difference in the prevalence of OCTI (*p* = 0.077). Age and clinical trachoma (*r* = −0.014; *p* < 0.001) or OCTI (*r* = −0.026; *p* = 0.031) prevalence were negatively correlated. Clinical trachoma was different in different districts and counties (*p* < 0.001). Trachoma warrants close attention in Shanghai migrant children because the condition remains endemic in some schools.

## 1. Introduction

Trachoma is a well-known infectious eye disease caused by serotypes A, B, Ba, and C of* Chlamydia trachomatis*. It is an endemic disease that shows an aggregated distribution in villages and communities within trachoma-prevalent areas throughout the world. Repeated infections lead to severe conjunctivitis, scarring of the eyelid, trichiasis, and corneal opacities that often result in visual impairment or blindness [1]. Trachoma was listed as second among five key preventable or treatable eye diseases by the World Health Organization (WHO) and was first in the infectious eye diseases category [2]. The WHO suggests implementing the SAFE strategy for eliminating blinding trachoma: surgery for trachomatous trichiasis, tetracycline, or azithromycin antibiotic treatment to clear ocular* C. trachomatis* infection (OCTI), facial cleanliness to reduce transmission of ocular* C. trachomatis*, and environmental improvement, particularly improved access to water and sanitation [2].

In mainland China, trachoma was previously widespread among the population with an average prevalence of 55% and trachoma-induced corneal opacity was the most frequent cause of blindness until the 1980s [3]. National programs for the prevention of trachoma, improved socioeconomic development, and advanced basic sanitation meant that the prevalence of trachoma declined rapidly. As of 2006, the results of the National Sample Survey on Disabilities in China revealed that blinding trachoma had moved to a lower rank on the list of the causes of blindness [4, 5]. However, the elimination of trachoma in any area of China has not been reported, and we suspect that trachoma remains endemic in some areas.

The epidemic condition of trachoma among the Shanghai migrant children, a rapidly expanding group, is of growing concern. In China internal migration is commonplace, as economic reforms have brought over 220 million migrants from villages to cities [6]. Most migrants travel from the western and central inlands, such as Anhui and Hunan, to the urban cities in the eastern coastal areas, such as Beijing and Shanghai [7]. In 2010, the number of migrants living in Shanghai reached 8.977 million, 38.21% of the total Shanghai resident population (23.475 million). Compared with the native Shanghai residents, the majority of these migrant labourers have lower incomes and lower levels of education [7]. The longstanding social system in China that separates rural and urban Chinese precludes many migrants from acquiring full citizenship in urban areas. As a result the migrant children attend migrant schools where the quality of teaching and facilities may be lower than in the schools the children of local origin attend [7]. Most of these migrant labourers live in crowded spaces with poor sanitary conditions that significantly increase the risk of poor health and infectious diseases in their children [8, 9].

In response to the need to understand the trachoma problem in migrant school aged children this study was commissioned by Shanghai Municipal Health Bureau, the government sector which is in charge of Shanghai health services and medical care management. From 1 December 2010 we conducted a survey to investigate the prevalence of trachoma and the correlating factors, the prevalence of OCTI and blindness caused by trachoma among Shanghai migrant children, and the results are documented and discussed in the present report. This report is the first of its kind in Shanghai and will provide important information for prevention and treatment of trachoma in children. This study was sponsored by Shanghai Charity Foundation, which further provided free antibiotics to the trachoma children.

## 2. Materials and Methods

### 2.1. Setting and Participants

Shanghai occupies a central location along China's eastern coastline. At the end of 2011, there were 17 administrative districts and counties in Shanghai. Approximately 10 to 20 residential communities or towns fall under the jurisdiction of each district or county. According to the data provided by the Shanghai Municipal Education Commission in 2010 (http://www.stats-sh.gov.cn/), a total of 154,265 children aged 6 to 16 years, most of whom are of the Han race, were studying in 206 migrant children schools (schools for children of rural-to-urban migration) near their parents' homes in Shanghai. These schools were located in nine administrative districts and one administrative county: Putuo, Minhang, Baoshan, Jiading, Jinshan, Songjiang, Qingpu, Fengxian, and Pudong Districts and Chongming County.

### 2.2. Study Design

This cross-sectional population-based survey was carried out between 1 December 2010 and 1 December 2012. The study personnel included 6 trained ophthalmologists and two experienced technicians from the Shanghai Eye Disease Prevention & Treatment Centre and 30 general practitioners from various districts in Shanghai. School officials, teachers, and students and their parents (guardians) were informed of the purpose and procedures of this survey 1 week before it was conducted.

The field survey was conducted in the appropriate areas for each school. The general practitioners investigated the toilet facilities and the availability of clean water (tap water) for the children to wash their hands and faces. The facial hygiene of each child was examined, and the practitioners collected birthdates, birthplace, previous eye disease diagnoses, and treatment history data from the schools. During the investigation, the general practitioners also watched the children's faces, and a “clean face” was defined as a face free of flies and ocular or nasal discharge [10]. The ophthalmologists conducted eye examinations. The distance visual acuity with the subject's usual visual correction, if any (here referred to as the presenting distance visual acuity) [11], was firstly measured using a Snellen E chart. If the presenting distance visual acuity was lower than 20/400 (defined as blindness) [11], the main cause of blindness was subsequently identified. All of the children were then given a basic eye surface examination consisting of an examination of the eyelid and cornea with a handheld slit-lamp biomicroscope at 2.5x magnification. The eyes were examined for the presence of trichiasis or corneal opacity, which was followed by eversion of the upper lid and inspection of any follicles, infiltrations, or scars in the conjunctiva. Trachoma was clinically diagnosed and assessed in accordance with the simplified classification for trachoma proposed by WHO in 1987, as follows [12]: trachomatous inflammation-follicular (TF) was defined by the presence of 5 or more follicles (diameter > 0.5 mm) in the central part of the upper tarsal conjunctiva. Trachomatous inflammation-intense (TI) was defined by pronounced inflammatory thickening of the upper tarsal conjunctiva with more than half of the normal deep tarsal vessels obscured; trachomatous scarring (TS) was defined by the presence of scarring in the tarsal conjunctiva accompanied by white lines or bands. Trachomatous trichiasis (TT) was defined by at least 1 eyelash rubbing on the eyeball or evidence of recent removal of in-turned eyelashes. Corneal opacity (CO) was defined by easily visible corneal opacity over the pupil in which the dense nature of the opacity obscured at least part of the pupil margin when viewed through the opacity or opacity resulting in significant visual impairment (i.e., worse than 20/60 vision).

Only children with a positive clinical trachoma evaluation, defined as any sign of trachoma according to the WHO simplified system [12], were then tested with a further latex immunochromatography test. A child was confirmed as an OCTI patient when both clinical and further latex immunochromatography tests were positive. Two experienced technicians collected conjunctival secretion samples from the eyes with positive clinical trachoma diagnoses by rolling sterile swabs over the conjunctiva at least 4 times. Then they conducted the latex immunochromatography test on the secretion samples with the* C. trachomatis* Rapid Test Device (ACON, Hangzhou, China) to detect* C. trachomatis*. Hurley and associates recently evaluated the performance of the ACON Chlamydia Rapid Test Device test (also used in the present study) compared with gold standard nucleic acid amplification testing, and they found that the sensitivity and specificity of the ACON were 43.8% and 98.3% in men and 66.7% and 91.3% in women, respectively, but this was in genital chlamydia, not ocular [13]. The ACON latex immunochromatography test was simple and inexpensive compared to a standard nucleic acid amplification test, and that is why we chose the ACON test for the present large-scale population-based survey. However, the limitation of the ACON latex immunochromatography test was obvious: with the chance of a high proportion of false-negatives, this test should not be used in clinical settings.

### 2.3. Quality Control

Before the formal investigation began, two experts in clinical trachoma diagnosis and treatment (Haidong Zou and Jianfeng Zhu) trained all of the study personnel. The training included descriptions of the purpose of the survey, the methodology of the survey, and the criteria for clinical trachoma diagnosis and grading. Standardised images for clinical trachoma diagnosis were used for training. Posttraining evaluation of the diagnostic accuracy was also undertaken by examinations to diagnose actual cases and those based on pictures of cases. We performed a preliminary investigation in four migrant children schools before the formal survey to examine the consistency of the diagnostic results between the different ophthalmologists. Overall, there was a high consistency between the two experts (*κ* = 0.93) in terms of trachoma diagnosis (prevalence rate). All of the problems encountered during this fieldwork were discussed and resolved by all study personnel. The study personnel leader reviewed suspected or confirmed positive cases. Any unresolved disagreements between the two assessors were referred to the group leader (Haidong Zou) for arbitration.

### 2.4. Ethics Statement

Because many of the migrant labourers work and live at building sites or bazaars and their children are placed in migrant children schools, it was difficult to obtain signed written consent from all of the parents during our investigation. With help from the school officials and teachers, oral informed consent to participate in this study was obtained from all the guardians (mostly by phone) and documented by the teachers. This research (including oral consent) was conducted according to the tenets of the Declaration of Helsinki and was approved by the Institutional Review Board at the Shanghai General Hospital, Shanghai Jiao Tong University.

### 2.5. Data Processing and Analysis

Experienced ophthalmic epidemiologists audited the completeness and logic of the data. The survey results were inputted, verified, and archived using the EpiData 3.0 program (The EpiData Association, Odense, Denmark). The statistical analyses were performed with the SPSS 16.0 software package (SPSS Inc., Chicago, IL, USA). Independent sample *t*-test was used to compare the age distributions across gender. Chi-square was used to compare the differences in the prevalence of clinical trachoma or OCTI among gender or different district and county distributions. Linear correlation was used to compare the differences in the prevalence of clinical trachoma or OCTI among age groups. *p* values < 0.05 were considered statistically significant.

## 3. Results

In all of 206 migrant children schools, there were at least two toilets available 24 hours and there was sufficient clean water (tap water) free-of-charge for the children to wash their hands and faces. A total of 153,977 students aged 6 to 16 years underwent interviews and eye examinations, which corresponds to a response rate of 99.8% (153,977/154,265). Of the participating students, 89,660 were male (58.2%), and 64,317 were female (41.8%) (Table 1). The average age of the students was 9.39 years with a standard deviation (SD) of 2.00 years. The average age of the male and female students was 9.39 years (SD: 1.99 years) and 9.38 years (SD: 2.01 years), respectively. The age distributions did not differ by gender (*t* = 1.15; *p* = 0.25). More than 99% of the children had clean faces at the time of the investigation. Presenting distance visual acuities worse than 20/400 were found in eight eyes from seven students. Six eyes were blind from congenital cataracts and two had macular dystrophy; none of the blindness cases was caused by trachoma.

### 3.1. Prevalence of and the Factors Correlated with Clinical Trachoma

Trachoma was clinically diagnosed in 8,029 cases, which yielded a prevalence of 5.2%. The total, male, and female patient numbers and prevalence rates of the different clinical trachoma classifications according to the WHO 1987 classification are shown in Table 1. The total, male, and female patient numbers and prevalence rates of clinical trachoma in the different age groups are shown in Table 2. The prevalence of clinical trachoma was higher among females than males (*χ* ^2^ = 66.9; *p* < 0.001). Negative linear correlations existed between age and the prevalence of clinical trachoma (*r* = −0.014; *p* < 0.001).

**Figure 1:**
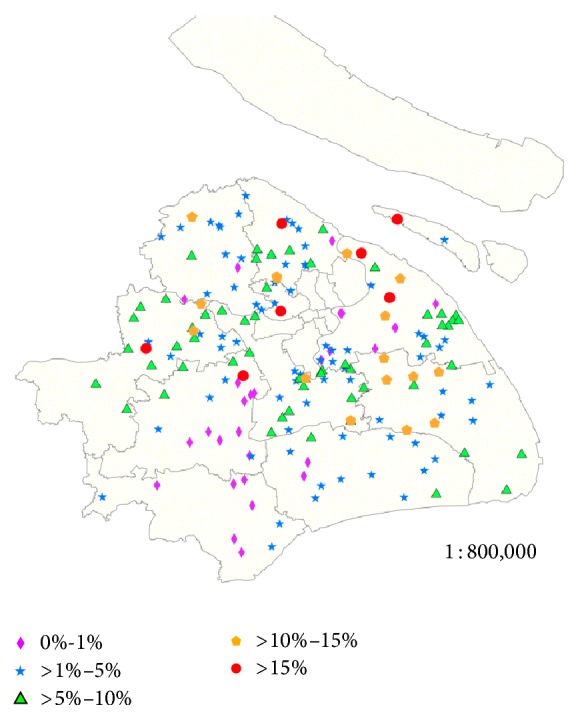
Geographical distribution of each individual migrant school with different prevalence of clinical trachoma in Shanghai. Symbols represent the prevalence according to the map key.

The 206 migrant children schools were geographically located in 10 districts. In each district, the prevalence of clinical trachoma among local migrant children was estimated as 1.3% to 8.7%. The number of migrant children schools and prevalence of clinical trachoma in different district and county distributions are shown in Table 3. In 6 schools (2.9%, 6/206), no students with clinical trachoma were confirmed. In 24 schools (11.7%, 24/206), the prevalence of clinical trachoma was between 0% and 1%. In 89 schools (43.2%, 89/206), the prevalence of clinical trachoma was between 1% and 5%. In 7 schools (3.4%, 7/206), the prevalence of clinical trachoma was more than 15%. The schools with the highest prevalence of clinical trachoma among the local migrant children were shown in Figure 1. Further analysis revealed a significant relationship between the district and county distributions and the prevalence of clinical trachoma among local migrant children (*χ* ^2^ = 714.0; *p* < 0.001).

### 3.2. Prevalence of and the Factors Correlated with Immunologically Confirmed OCTI

Among the 8,029 clinical trachoma cases, conjunctival secretion samples were collected from 6,823 (85.0%) students, including 3,642 males and 3,181 females. The other 1,206 students failed to cooperate with the sample collection because they were unable to tolerate the sterile swabs rolling over the conjunctiva. The latex immunochromatography tests revealed that 2,073 (30.4%) of the 6,823 students were* C. trachomatis* positive. Bilateral infections occurred in 1,602 students and monocular infections occurred in 471 students. The total, male, and female patient numbers and prevalence of OCTI in the different age groups are shown in Table 4. The prevalence of OCTI was higher among females than males, but there was not statistical difference (*χ* ^2^ = 3.131; *p* = 0.077). Negative linear correlations existed between age and the prevalence of OCTI (*r* = −0.026; *p* = 0.031).

The number of migrant children schools and prevalence rates of OCTI in different district and county distributions are shown in Table 5. In each district, the prevalence rate of OCTI among local migrant children was a majority of greater than 10% (Table 5). Further analysis revealed a statistically significant difference with district and county distributions and the prevalence of OCTI among local migrant children (*χ* ^2^ = 165.3; *p* < 0.001).

## 4. Discussion

The prevalence of trachoma in children can represent the burden of trachoma in a specific area [14]. As any child under 16 should receive 9 years of compulsory education in China, in Shanghai, a vast majority of the migrant labourers' children receive education free-of-charge in the nearby migrant children schools. Because our previous report found no cases of blindness caused by trachoma in children of local origin more than 6 years old in Shanghai [15], a positive conclusion can be drawn that there is no blindness caused by trachoma in children aged 6–16 in Shanghai, regardless of whether they are native or migrant. It is very likely that blindness from trachoma mostly occurs much later in life after repeated* C. trachomatis* infections [2].

Previous studies in China have only focused on the prevalence of clinical trachoma, which was much higher than in our study [16, 17]. The differences might be attributable to the fact that the previous cluster sampling designed studies were conducted in relatively small samples of rural Chinese children. However, we found that trachoma is still endemic in some migrant children schools, which implies that trachoma warrants close attention in Shanghai because these infected children and their close contacts face a high risk of repeated infections.

To further realize the public health risks of* C. trachomatis* infection in Shanghai, we investigated the prevalence of OCTI confirmed immunologically in the migrant children in school. To the best of our knowledge, this is the first report of a large-scale population-based prevalence rate of OCTI in Chinese children. We think that the latex immunochromatography test positive rate (30.4%) was likely to be because trachomatous follicles persist for long periods of time even when the infection has vanished, and latex immunochromatography requires a high antigen content, so false-negative diagnoses might have occurred in patients with subtle* C. trachomatis* infections.

Trachoma is a global problem [18]. Frequently reported risk factors that may be related to the prevalence of active trachoma include poor living conditions, heavy wind and dust, dirty water or no water, and elevated fly (*Musca sorbens*) densities [16, 17, 19–22]. We found no significant correlation between any of these factors and the prevalence of clinical trachoma or OCTI that was confirmed immunologically. The schools observed provided sufficient clean water to help the children maintain clean faces and hands. Future close observations of the living conditions, physical fitness, hygiene habits, and susceptibility to* C. trachomatis* infections might help elucidate the exact risk factors of OCTI in Shanghai migrant school children. The prevalence in this study was 30.4% in these migrant school aged children. WHO guidelines are that a prevalence of TF of ≥5% in 1–9-year-old children indicates that active trachoma is a public health problem, and in such areas the A, F, and E components of the SAFE strategy should be implemented including mass drug administration of the antibiotic azithromycin [12]. In many other countries worldwide the prevalence in some areas reaches more than 10% including many countries in Africa [23–31]. Even in a developed country such as Australia, some indigenous populations in remote areas have a prevalence of trachoma above WHO's elimination threshold [32].

Most studies find no gender differences in prevalence of active trachoma [20, 33]. But the prevalence of trachoma was previously found to be much higher in girls than in boys in China [17], which is consistent with our study results, and has also been shown in Cameroon [28]. However, in contrast a study in Guinea Bissau found that being female was associated with reduced odds of active trachoma, but not infection [34]. The authors of the Chinese study suggested that, in rural Chinese areas, male children experience better living conditions than female children due to the traditional preference for males and this is found in other communities [17, 35]. It has also been suggested that female children may be at greater risk for active trachoma because of their role in taking care of younger children in the family [20]. Like previous studies [36, 37], the present study observed a significant difference of the prevalence of trachoma with age. Older children and adolescents have greater understanding of human health and better hygiene habits, and we hypothesize that these might lead to decreases in the prevalence of trachoma. In addition, the duration of episodes of active disease and infection decrease with increasing age [38–40].

There are inherent weaknesses of this study. Trachoma is supposed to be more common among preschool children than older children. However, we found it almost impossible to conduct a survey of preschool migrant children in Shanghai. Most of the preschool migrant children live with their parents in tens of thousands of workplaces with no exact demographic data. In some cases, the preschool children live with grandparents who do not live in Shanghai because of the difficulty involved in looking after them while the parents work. Infants also cannot cooperate with the eye examination and conjunctival secretion sample collection. An African study suggested that school surveys might underestimate the prevalence because differences were found between those who attended school and those who did not; however, in Nigeria where school attendance is high the differences were not seen [38, 39]. As the rate of school attendance is high in the Shanghai migrant school aged children the prevalence in our study may be fairly accurate. Because it was impossible to collect conjunctival secretion samples from all 153,977 students, we only collected samples from those with distinct clinical features, and a tiny group of children with immediate early infection and no signs of trachomatous follicles might have been omitted. Some students would not cooperate with having a swab. We presumed that 30.4% of the uncooperative 1206 students were also likely to be positive as this was the rate in those who could be swabbed; then a total of 2439 cases of the 8029 clinical trachoma students would be OCTI positive, and in that situation the estimated OCTI prevalence would be 1.6%. Furthermore, as reported by Hurley and associates, although the latex immunochromatography kit used in the present study has high specificity between 33.3% and 57.2% of true OCTI infected cases will not be positive compared to the rates by gold standard nucleic acid amplification tests [13]. Accurate detection of serotypes A, B, Ba, and C* C. trachomatis* requires a more dedicated latex immunochromatography kit or a combination of the current kit with a highly sensitive nucleic acid amplification based detection method; this might be possible in future studies. We also did not collect information from children of local origin in nonmigrant schools as there are no recent data on the prevalence of trachoma in native Shanghai children; this would have provided important information and enabled comparisons with the rates in the native population.

## 5. Conclusion

The prevalence of clinical trachoma was 5.2% and the infection was somewhat prevalent (prevalence close to or higher than 5% in some migrant schools). Given its large population, China has a large number of children who are currently living in environments similar to, or worse than, those of the migrant school children in Shanghai. This indicates that trachoma should still be regarded as an issue of concern in China. Mainly based on the present study results, on March 5, 2013, Shanghai Municipal Health Bureau issued a project plan in order to eliminate blinding trachoma and to reduce the active trachoma in Shanghai before 2016, which is in line with the Chinese national program.

## Figures and Tables

**Table 1:** Prevalence of different clinical trachoma classifications in 153,977 migrant school aged students in Shanghai by gender.

Trachoma classification^#^, *n* (%)	Total *n* = 153977	Male *n* = 89660	Female *n* = 64317
TF	7878	4238	3640
TI	69	37	32
TS	78	46	32
TT	1	0	1
CO	3	2	1

Total	8029 (5.2)	4323 (4.8)	3706 (5.8)

*Note*. ^#^Trachoma classification according to WHO 1987. TF: trachomatous inflammation-follicular; TI: trachomatous inflammation-intense; TS: trachomatous scarring; TT: trachomatous trichiasis; CO: corneal opacity.

**Table 2:** Age and gender distributions of clinical trachoma in the 153,977 migrant school aged students in Shanghai.

Age (year)	Male students		Female students		Total students	
	Total number	Children with trachoma, *n* (%)	Total number	Children with trachoma, *n* (%)	Total number	Children with trachoma, *n* (%)
6	3194	168 (5.3)	2407	146 (6.1)	5601	314 (5.6)
7	13648	706 (5.2)	9813	568 (5.8)	23461	1274 (5.4)
8	17746	839 (4.7)	12861	750 (5.8)	30607	1589 (5.2)
9	14664	739 (5.0)	10559	637 (6.0)	25223	1376 (5.5)
10	14137	715 (5.0)	10146	625 (6.2)	24283	1340 (5.5)
11	12831	551 (4.3)	9079	530 (5.8)	21910	1081 (5.0)
12	7846	386 (5.0)	5314	297 (5.6)	13160	683 (5.2)
13	2997	111 (3.7)	2025	102 (5.0)	5022	213 (4.2)
14	1395	54 (3.9)	1095	25 (2.3)	2490	79 (3.2)
15	796	34 (4.3)	683	20 (2.9)	1479	54 (3.7)
16	406	20 (5.0)	335	6 (1.8)	741	26 (3.5)

Total	89660	4324 (4.8)	64317	3706 (5.8)	153977	8029 (5.2)

**Table 3:** Different district and county distributions of clinical trachoma in the 153,977 migrant school aged children in Shanghai.

District or county name	Population of children in migrant school	Children with trachoma, *n* (%)	Number of migrant children schools	0%-1%	>1%–5%	>5%–10%	>10%
Pudong	36563	2314 (6.3)	59	7	19	23	10
Fengxian	12623	395 (3.1)	16	2	11	3	0
Jiading	16374	449 (2.7)	19	2	15	1	1
Songjiang	20346	1118 (5.5)	20	9	5	4	2
Jinshan	4732	63 (1.3)	11	7	4	0	0
Putuo	1826	137 (7.5)	3	0	1	1	1
Baoshan	25057	1244 (5.0)	27	2	15	7	3
Minhang	20305	1156 (5.7)	25	0	10	13	2
Chongming	1470	128 (8.7)	2	0	1	0	1
Qingpu	14681	1025 (7.0)	24	1	8	10	5

Total	153977	8029 (5.2)	206	30	89	62	25

**Table 4:** Age and gender distributions of ocular *Chlamydia trachomatis* infection in 6,823 migrant school aged students in Shanghai.

Age (year)	Male students		Female students		Total students	
	Total number	Ocular *C. trachomatis* infection, *n* (%)	Total number	Ocular *C. trachomatis* infection, *n* (%)	Total number	Ocular *C. trachomatis* infection, *n* (%)
6	130	35 (26.9)	111	39 (35.1)	241	74 (30.7)
7	548	171 (31.2)	421	134 (31.8)	969	305 (31.5)
8	691	208 (30.1)	661	224 (33.9)	1352	432 (32)
9	631	185 (29.3)	542	168 (31)	1173	353 (30.1)
10	622	191 (30.7)	550	171 (31.1)	1172	362 (30.9)
11	486	142 (29.2)	475	140 (29.5)	961	282 (29.3)
12	333	82 (24.6)	279	84 (30.1)	612	166 (27.1)
13	91	32 (35.2)	87	28 (32.2)	178	60 (33.7)
14	54	14 (25.9)	32	6 (18.8)	86	20 (23.3)
15	34	8 (23.5)	18	5 (27.8)	52	13 (25)
16	22	5 (22.7)	5	1 (20)	27	6 (22.2)

Total	3642	1073 (29.5)	3181	1000 (31.4)	6823	2073 (30.4)

**Table 5:** Different district and county distributions of ocular *C. trachomatis* infection in 6,823 migrant school aged students in Shanghai.

District or county name	Ocular *C. trachomatis* infection, *n* (%)	Number of migrant children schools	Number of schools with different prevalence of ocular *C. trachomatis *infection confirmed immunologically	
			<10%	≥10%
Pudong	632 (28.8)	59	9	50
Fengxian	177 (49.0)	16	0	16
Jiading	153 (35.7)	19	0	19
Songjiang	192 (41.0)	20	12	8
Jinshan	12 (41.4)	11	8	3
Putuo	29 (22.7)	3	0	3
Baoshan	201 (19.6)	27	5	22
Minhang	300 (27.8)	25	1	24
Chongming	38 (30.9)	2	2	0
Qingpu	339 (34.3)	24	1	23

Sum	2073 (30.4)	206	38	168
